# Audit on Blood Tests Performed by Crisis Resolution and Home Treatment Team North East Kent (NEKCRHTT): Clinical Indications, Current Practice and Recommendations

**DOI:** 10.1192/bjo.2025.10681

**Published:** 2025-06-20

**Authors:** Sabaritha Subramania Pillai, Carolina Pressanto, Victoria Clark

**Affiliations:** Kent and Medway NHS and Socialcare Partnership Trust, Kent, United Kingdom

## Abstract

**Aims:** This audit project aims to assess whether the Crisis Resolution and Home Treatment Team North East Kent (NEKCRHTT) is following the trust protocols for requesting blood tests, or according to clinical indication.

**Methods:** For the first cycle, data collection was done from 1 August to 31 October 2023 from all clients under NEKCRHTT who had undergone blood tests and were under psychotropics. Information regarding documentation of clinical indication and which investigations were requested was collated and checked for compliance with trust policy. For the re-audit cycle, data collection was done from 1 July to 31 September 2024 and results were compared for improvements in adherence to the trust policy.

**Results:** First cycle results showed that the trust policy was not followed in 33.3% cases, in which mostly TFT, folate and B12 levels were requested without clear documentation of clinical indication. Results were presented with following recommendations: To have the trust policy poster easily accessible in clinical areas; prescribers to be aware of the policy and follow guidelines when appropriate; if there is clinical indication that deviates from policy, for this to be clearly documented on client’s notes. The re-audit cycle results showed an improvement in adherence to the trust policy: only 13.3% cases did not follow the policy. Documentation of rationale for requesting blood tests has also improved. When deviating from policy, it is still mostly by requesting TFT, B12 and folate, although the frequency has reduced.

**Conclusion:** The use of blood tests in a mental health crisis can serve many purposes, such as identifying potential damage secondary to overdose, aid in the differential diagnosis when considering organic causes for current presentation, ascertain renal/liver function before prescribing a new medication, among others. Having blood tests done according to the clinical indication and following a clear protocol assures accuracy in the management of the results. The implementation of change has resulted in improvement in adherence to the trust policy and the documentation of rationale for requesting investigations. This will hopefully assure accuracy in the management of the results, as well as avoid confusion of incidental findings.

